# Intestinal tuberculosis in a child: a case report

**DOI:** 10.3389/fmed.2026.1828935

**Published:** 2026-05-04

**Authors:** Can Guo, Yu Guo, Guizeng Zhao, Min Zang, Linyan Yao, Yuhua He, Yanjing Pan, Weihao Wu, Yu Pang, Junwei Cui

**Affiliations:** 1 Department of Tuberculosis, The First Affiliated Hospital of Henan Medical University, Xinxiang, China; 2 Health Management Center, The First Affiliated Hospital of Henan Medical University, Xinxiang, China; 3 Department of Geriatrics, The First Affiliated Hospital of Henan Medical University, Xinxiang, China; 4 Department of Radiation, The First Affiliated Hospital of Henan Medical University, Xinxiang, China; 5 Department of Bacteriology and Immunology, Beijing Chest Hospital, Capital Medical University, Beijing, China

**Keywords:** bowel obstruction, diagnosis, intestinal tuberculosis, pediatric, surgical treatment

## Abstract

Tuberculosis (TB) is an infectious disease and one of the major causes of mortality worldwide, especially in children under the age of 15 years. Abdominal TB includes TB of bowel, peritoneum, lymph nodes, or other solid organs, with a predominance of intestinal and peritoneal forms of the disease. Most pediatric cases may be misdiagnosed due to subclinical symptoms. This case report describes an 11-year-old boy diagnosed with intestinal tuberculosis (ITB) complicated by bowel obstruction. No prior recommendation for surgical therapy resulted in a significant complication of enterobiasis. ITB is a challenging infectious disease associated with high mortality rates, particularly in the pediatric population. This case study emphasizes the importance of pediatric ITB surgery to overcome unfavorable consequences.

## Introduction

Tuberculosis (TB) is an infectious disease caused by *Mycobacterium tuberculosis* (MTB). It is the second leading infectious cause of death worldwide, with an estimated 1.23 million deaths (196,800 among children) in the past year, according to the Global Tuberculosis Report 2025 by the World Health Organization (WHO) (1). Among all pediatric TB cases, 10% are extrapulmonary, and 1–3% involve ITB, particularly in children under 15 years of age (2). ITB may occur in isolation or in association with pulmonary tuberculosis (3). Pediatric ITB cases require simultaneous anti-TB therapy and surgery, as complications can occur, such as bowel perforation, obstruction, fistula formation, abscesses, and hemorrhage (4, 5). ITB is rare in children and usually spreads to the peritoneum or gastrointestinal tract (6). The clinical symptoms of ITB tend to be indeterminate and nonspecific, leading to delayed diagnosis and increased mortality (7). In this study, we present a case of ITB complicated by bowel obstruction, for which surgery and anti-tuberculosis treatment (ATT) are recommended. This unique case emphasizes the importance of surgery and therapy for children with ITB who are at risk of bowel obstruction.

## Case presentation

An 11-year-old boy initially presented with a one-month history of intermittent abdominal distention and fever. A significant unintentional weight loss of up to 5 kg was observed. When the symptoms initially appeared, he pursued local medical treatment without clinical improvement. He continued to experience abdominal distention with episodes of fever. He was treated with anti-infective therapy, as local doctors diagnosed his illness as enteritis. However, the therapeutic effect was poor. Upon physical examination, he appeared clinically ill, with a fever of 39 °C and visible wasting. No symptoms such as lymphadenopathy or jaundice were observed. His vital signs were within normal limits. Abdominal examination revealed moderate distension with no tenderness on percussion. He was a local primary school student with no travel history outside China. His grandmother had a history of pulmonary TB.

His laboratory results demonstrated a low hemoglobin level (79 g/L) and no leukocytosis. There was an elevation in C-reactive protein levels (18.53 mg/L). His tuberculosis antibody test (serum IgG), tuberculin skin test (TST), and T-SPOT.TB (T-SPOT, IGRA) were positive. Erythrocyte sedimentation rate increased (42 mm/h). However, tests for Aspergillus galactomannan, β-D-glucan, respiratory viruses, tumor markers, autoimmune disease antibodies, and enzyme-linked immunosorbent assay for human immunodeficiency virus were negative. Sputum smear, sputum culture, and sputum GeneXpert MTB/RIF tests were negative (Table 1). The chest computed tomography (CT) demonstrated no obvious abnormality. An abdominal ultrasound revealed bowel narrowing.

**Table 1:** Core laboratory findings of the patient.

Laboratory findings	Results
Hemoglobin	79 g/L
C-reactive protein	18.53 mg/L
Erythrocyte sedimentation rate	42 mm/h
Tuberculosis antibody test (serum IgG)	Positive
Tuberculin skin test (TST)	Positive
T-SPOT.TB(IGRA)	Positive
Aspergillus galactomannan	Negative
β-D-glucan	Negative
Respiratory viruses	Negative
Tumor markers	Negative
Autoimmune disease antibodies	Negative
Enzyme-linked immunosorbent assay for human immunodeficiency virus	Negative
Sputum smear	Negative
Sputum culture	Negative
Sputum GeneXpert MTB/RIF tests	Negative

**Figure 1:**
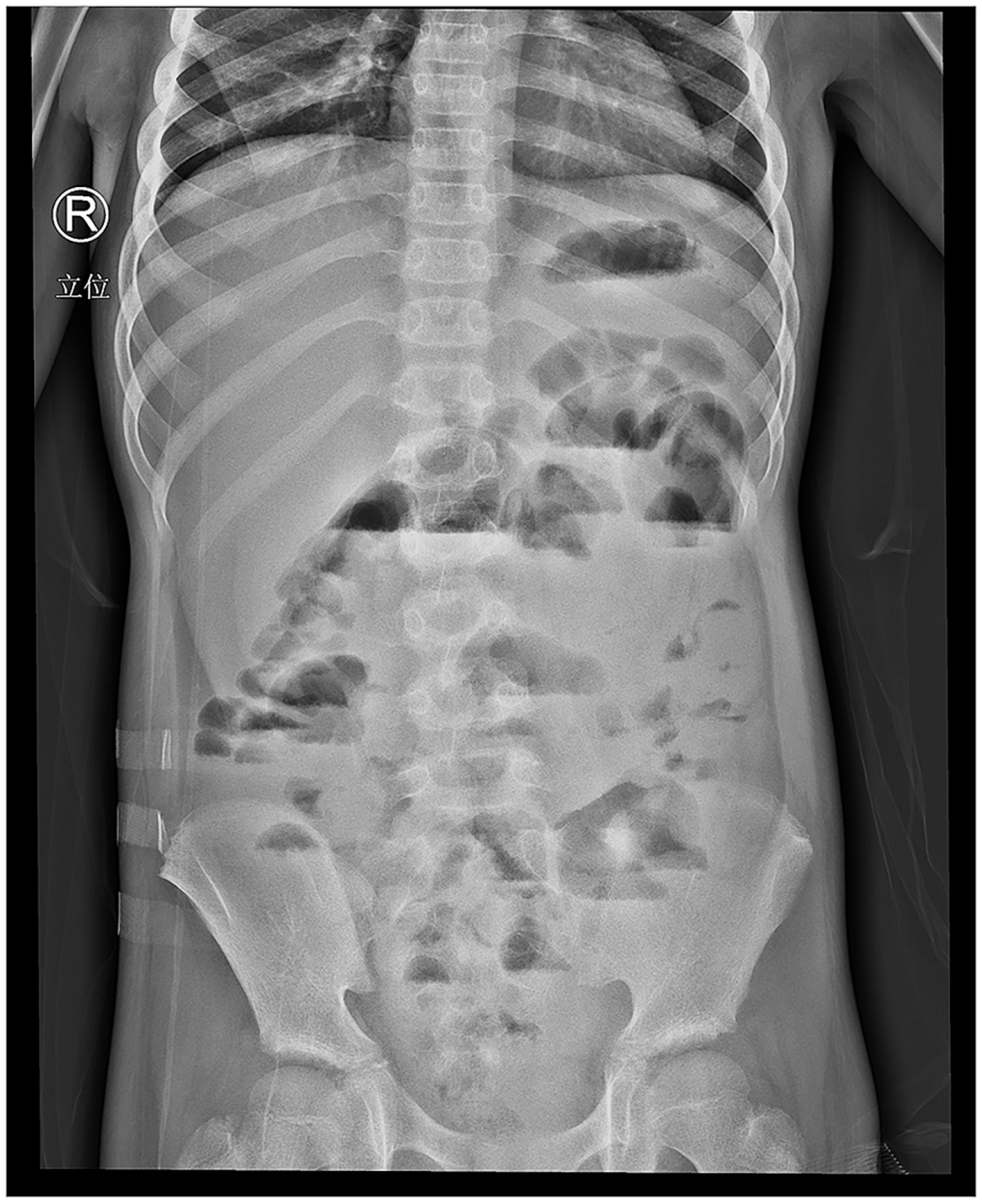
Abdominal X-ray examination revealed intestinal obstruction. The middle and upper abdominal parts of the intestine revealed gas accumulation, expansion, and multiple liquid–gas planes with different heights.

On the other hand, the abdominal X-ray demonstrated gas accumulation, distension, and multiple air-fluid levels of varying heights in the middle and upper abdominal segments of the intestine. The patient was diagnosed with bowel obstruction (Figure 1). At that time, several differential diagnoses were considered, including extrapulmonary tuberculosis, inflammatory bowel disease, yeast and fungal infections, and primary or metastatic malignancy. As the patient had a history of contact with individuals with TB, he was more likely to be diagnosed with ITB. Initially, a 4-drug regimen (daily standard weight-based dosing of rifampicin [450 mg, 20 mg/kg], ethambutol [750 mg, 25 mg/kg], isoniazid [300 mg, 15 mg/kg], and pyrazinamide [1,250 mg, 40 mg/kg]) was prescribed for suspected ITB.

**Figure 2:**
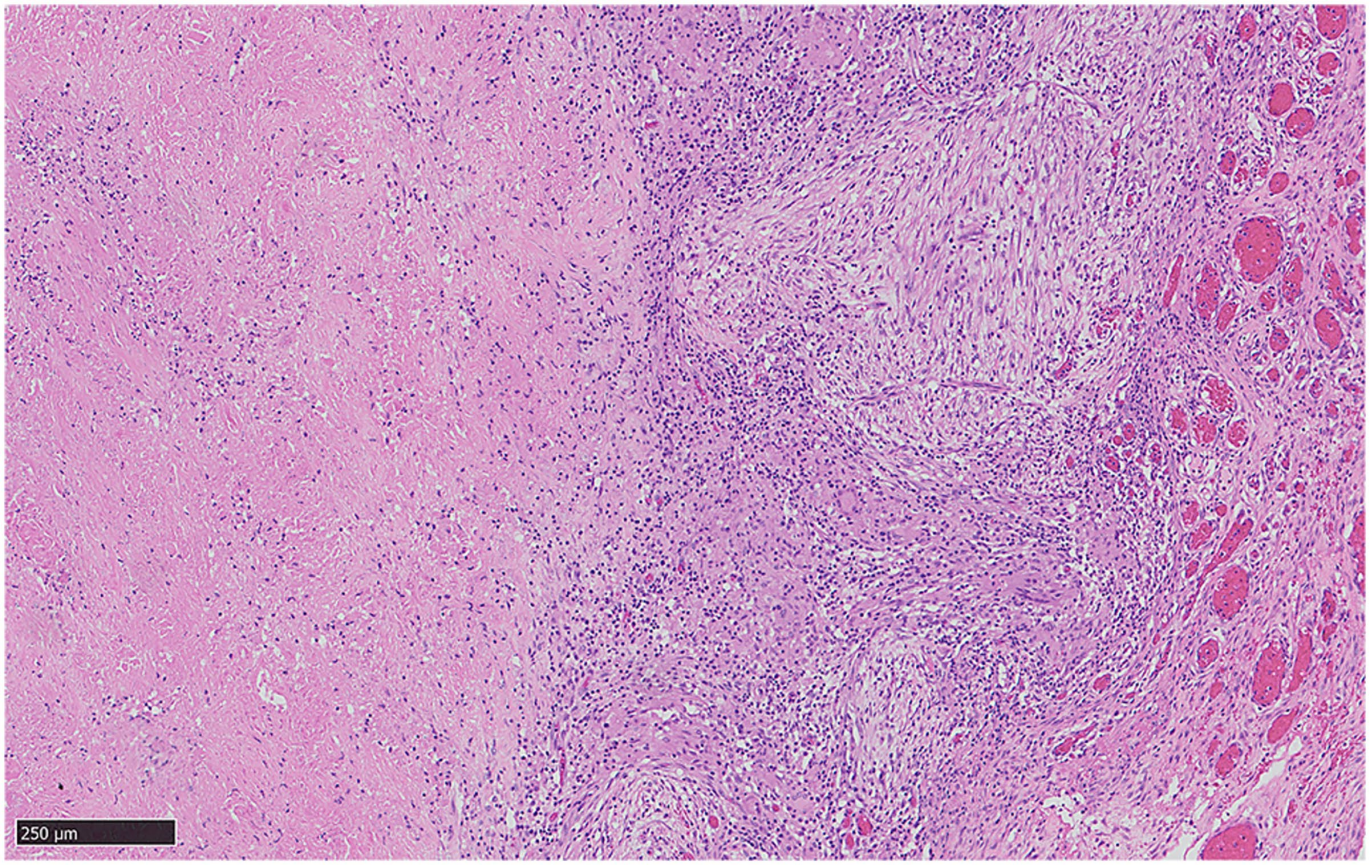
Pathological results were consistent with TB. Pathological results revealed caseous granulomas, multinucleated giant cells, and epithelial-like cell infiltration consistent with TB. Molecular pathology results demonstrated TB-DNA positive.

One week after the diagnosis of ITB and initiation of anti-tuberculosis treatment, the patient’s ileus symptoms worsened (Table 2). Laparotomy was performed on the pediatric patient, and the intestine was widely adherent. The intestinal wall and mesentery were covered with caseous granulomas. The intestinal wall was necrotic and perforated. The pediatric patient underwent intestinal adhesiolysis, fistula repair, and suture repair. The patient missed the optimal time for surgery due to misdiagnosis. After surgery, the patient was admitted to the pediatric intensive care unit for postoperative management. Pathological results revealed caseous granulomas, multinucleated giant cells, and epithelioid cell infiltration, consistent with TB (Figure 2). Molecular pathology results revealed that the patient was TB-DNA positive. The patient’s clinical symptoms improved following treatment. Neither cholestasis nor liver dysfunction from the combination of short bowel syndrome and TB drug toxicity occurred in the patient. His anti-tuberculosis treatment was continued after the operation. He continued TB therapy for a planned course of 9 months.

**Table 2:** Treatment timeline and clinical symptoms of the patient.

Timeline and Symptoms	Symptom onset	Initial misdiagnosis	Initiation of ATT	Clinical deterioration	Surgical intervention
Timeline	Jun 1	Jun 20	July 3	July 11	July 11
Symptoms					
Abd distention	Yes	Yes	Yes	Yes	Yes
Abd pain	No	No	No	Yes	Yes
Fever	Yes	Yes	Yes	Yes	Yes
Diarrhea	No	No	No	No	No
Constipation	No	No	No	No	No
Others	No	No	No	No	No

ATT, anti-tuberculosis therapy; ABD, abdominal.

## Discussion

TB, especially ITB, remains a challenging infectious disease. It is a chronic, specific infection caused by *Mycobacterium tuberculosis*, accounting for 1–3% of all TB cases (8). Diagnosis often requires confirmation through tissue cultures and pathological examinations. ITB is considered rare in children and adolescents but is relatively common in adults (9). Nonspecific clinical and laboratory features of ITB can cause delays in diagnosis and initiation of treatment. A triad of abdominal distension, fever, and weight loss may be suggestive of ITB, as observed in this case. The diagnostic challenge is further illustrated by cases in which ITB mimics malignancy. For instance, gastrointestinal basidiobolomycosis, a rare fungal infection, can mimic both ITB and colonic malignancy, underscoring the importance of tissue diagnosis to differentiate these conditions (10). Similarly, cases of concurrent ITB and adenocarcinoma have been reported, highlighting the need for clinicians to consider the possibility of coexisting conditions when faced with atypical presentations (11). It also has been reported that a case of miliary TB in a 2-year-old child who only presented with a history of constipation, weight loss, marked abdominal distension with hepatosplenomegaly (12). These examples demonstrate the necessity for a multidisciplinary approach and the use of comprehensive diagnostic strategies to ensure accurate diagnosis and effective management of ITB, particularly in differentiating it from inflammatory bowel disease and malignancies. ITB should be considered as a differential diagnosis, especially in endemic areas, to achieve an early diagnosis. ITB can lead to severe complications, including the formation of abscesses, fistulas, strictures, perforations, and obstructions (13). ITB can result in systemic sepsis and even death if not treated promptly (14). Although ITB is more common in immunocompromised individuals, serious complications can also occur in immunocompetent individuals (15). Delayed diagnosis of ITB has been associated with an increased risk of complications (16). In this study, a lack of early recognition presumably contributed to the development of severe complications. Surgery is essential for patients presenting with intestinal obstruction or perforation.

Although contrast-enhanced CT could reveal several features such as bowel wall thickening and mesenteric lymphadenopathy, these findings are not pathognomonic for ITB (17). Abdominal X-ray can also detect changes within the intestinal wall and complications such as obstruction and perforation, making it a conventional and simple diagnostic tool for intestinal tuberculosis. In cases of diagnostic uncertainty, surgery may increase the probability of early diagnosis (3). Previously, a 41-year-old immunocompetent male was also diagnosed with intestinal tuberculosis through surgery (18). Although surgery is an important approach for ITB with severe complications, pharmacological anti-tubercular therapy is the essential component of treatment. The pediatric ITB regimen includes medications such as isoniazid, rifampicin, pyrazinamide, and ethambutol (15). Anti-tuberculosis treatment was initiated immediately after surgery. The duration of treatment varies among patients. To ensure complete eradication of ITB, the patient was prescribed a long course of ATT lasting for 9 months. The extended treatment duration aims to effectively eliminate TB infection and prevent the development of drug resistance. Recent studies provide insights which emphasize the importance of tailored approaches to improve outcomes. One significant consideration in ITB treatment is the optimal duration of therapy. A study conducted in Taiwan explored the impact of extending ATT from 6 to 9 months in patients with diabetes mellitus, finding that a longer treatment duration was associated with a lower recurrence rate, particularly when treatment was not supervised (19). This suggests that extending treatment duration could be beneficial in reducing recurrence rates in specific patient populations, aligning with the need for individualized treatment plans. Although pharmacological anti-tuberculosis treatment can treat most patients with ITB, surgical treatment is necessary when severe complications such as intestinal obstruction, intestinal adhesions, and intestinal degeneration arise as a result of the infection (20). In patients with ITB who develop severe complications, surgery should be performed as early as possible (21). Simultaneously, attention should be paid to the occurrence of postoperative complications. Postoperative complications of ITB mainly include anastomotic leakage, abdominal infection, malnutrition, and TB dissemination. It is necessary to observe post-surgery symptoms, standardize the anti-tuberculosis treatment, improve nutritional support, and conduct regular follow-up to reduce the risk of complications. The timing of surgical intervention in ITB is a critical decision point, particularly when symptoms worsen after the initiation of ATT. The complexity of ITB, characterized by its potential to mimic other gastrointestinal disorders such as Crohn’s disease and malignancy, necessitates a nuanced approach to management. The decision to proceed with surgery is often influenced by the persistence or exacerbation of symptoms despite ATT, as well as the presence of complications such as strictures or perforations. A study investigating clinical factors associated with surgical interventions in patients with intestinal obstruction caused by abdominal tuberculosis highlights the importance of imaging findings and the response to ATT in determining the need for surgery (22). This suggests that imaging and therapeutic response are pivotal in surgical decision-making. Similarly, another study emphasizes that intestinal strictures in ITB patients often show a poor response to ATT, with only a minority achieving stricture resolution, thereby necessitating surgical intervention in many cases (23). Moreover, the occurrence of paradoxical reactions, such as immune reconstitution inflammatory syndrome (IRIS), can complicate the clinical picture, leading to worsening symptoms after ATT initiation. A case study on TB-IRIS highlights the challenges in distinguishing between true disease progression and paradoxical inflammatory responses, which can influence the timing and necessity of surgical intervention (24). Similarly, the development of tuberculous intestinal perforation during ATT, as reported in another case, illustrates the potential for acute complications that necessitate prompt surgical management (25). The timing of surgical intervention in ITB is a multifaceted decision that hinges on the interplay between clinical presentation, imaging findings, and response to ATT. Persistent or worsening symptoms, particularly in the context of complications like strictures or perforations, often necessitate surgical evaluation. Acute peritonitis, intestinal obstruction, and intestinal perforation are the main clinical presentations requiring surgical intervention. In another case study, patients with ITB and severe complications were immediately treated with surgery (18). Surgical delay can aggravate the development of various complications in patients (16). In our report, the child developed severe complications due to surgical delay. After surgical treatment, the patient’s symptoms were improved. As a significant public health concern worldwide, ITB may affect virtually every organ system. A high index of clinical suspicion and recognition of consolidated complications of ITB require a timely surgical treatment, especially in high-risk populations such as pediatric patients (26).

## Conclusion

The present report describes a rare case of intestinal obstruction resulting from ITB. Acute peritonitis, intestinal obstruction, and intestinal perforation are the main clinical presentations requiring surgical intervention. ITB is associated with high mortality rates, especially in the pediatric population. This study emphasizes the importance of surgery in pediatric ITB patients to overcome unfavorable complications. Treatment options for ITB remain extremely limited. Therefore, the WHO needs to consider including parenteral anti-TB regimens for ITB in its official guidelines, particularly for pediatric patients.

## Data Availability

The raw data supporting the conclusions of this article will be made available by the authors, without undue reservation.
